# Supplementary figures and images for: Revealing the Meissner Corpuscles in Human Glabrous Skin Using In Vivo Non-Invasive Imaging Techniques
Source: Int J Mol Sci. 2023 Apr 12;24(8):7121. doi: 10.3390/ijms24087121 (PMC10138989; doi:10.3390/ijms24087121)

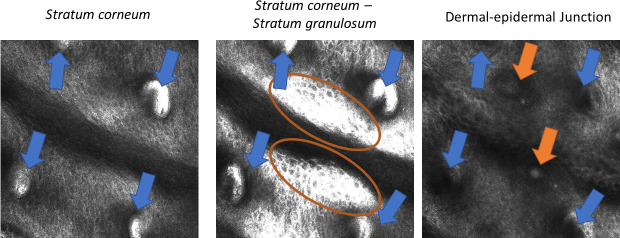

Supplement: Supplementary file 1 [file ijms-24-07121-s001.zip › Supplementary Figure S1.tif]

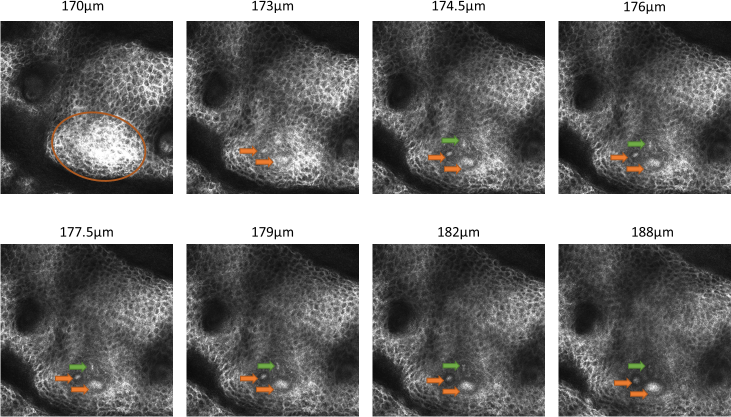

Supplement: Supplementary file 1 [file ijms-24-07121-s001.zip › Supplementary Figure S2.tif]

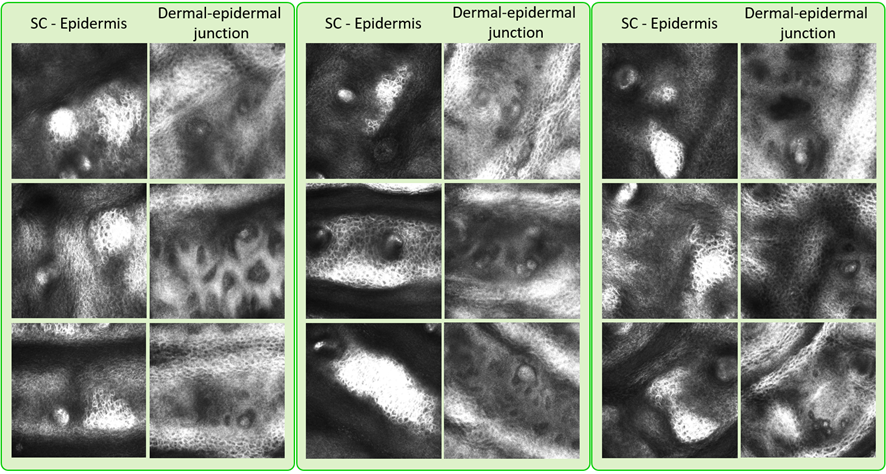

Supplement: Supplementary file 1 [file ijms-24-07121-s001.zip › Supplementary Figure S3.tiff]
